# Smartphone-Based Device for Colorimetric Detection of MicroRNA Biomarkers Using Nanoparticle-Based Assay

**DOI:** 10.3390/s21238044

**Published:** 2021-12-01

**Authors:** Tushar Krishnan, Hsin-Neng Wang, Tuan Vo-Dinh

**Affiliations:** 1 Fitzpatrick Institute for Photonics, Duke University, Durham, NC 27708, USA; tusharkrishnan@gmail.com (T.K.); hsinneng.wang@duke.edu (H.-N.W.); 2 Department of Biomedical Engineering, Duke University, Durham, NC 27708, USA; 3 Department of Chemistry, Duke University, Durham, NC 27708, USA

**Keywords:** smartphone-based device, colorimetric analysis, miRNA detection, medical diagnostics, cancer detection, biofuel research

## Abstract

The detection of microRNAs (miRNAs) is emerging as a clinically important tool for the non-invasive detection of a wide variety of diseases ranging from cancers and cardiovascular illnesses to infectious diseases. Over the years, miRNA detection schemes have become accessible to clinicians, but they still require sophisticated and bulky laboratory equipment and trained personnel to operate. The exceptional computing ability and ease of use of modern smartphones coupled with fieldable optical detection technologies can provide a useful and portable alternative to these laboratory systems. Herein, we present the development of a smartphone-based device called Krometriks, which is capable of simple and rapid colorimetric detection of microRNA (miRNAs) using a nanoparticle-based assay. The device consists of a smartphone, a 3D printed accessory, and a custom-built dedicated mobile app. We illustrate the utility of Krometriks for the detection of an important miRNA disease biomarker, miR-21, using a nanoplasmonics-based assay developed by our group. We show that Krometriks can detect miRNA down to nanomolar concentrations with detection results comparable to a laboratory-based benchtop spectrophotometer. With slight changes to the accessory design, Krometriks can be made compatible with different types of smartphone models and specifications. Thus, the Krometriks device offers a practical colorimetric platform that has the potential to provide accessible and affordable miRNA diagnostics for point-of-care and field applications in low-resource settings.

## 1. Introduction

MicroRNAs (miRNAs) are short RNA molecules, consisting of 18–25 nucleotides. They bind via hybridization to sequences in the untranslated regions of target messenger RNA (mRNA) molecules, which allows them to act as post-transcription gene expression regulators [1]. As miRNA levels affect mRNA translation and degradation, their dysregulation is often detected in various diseases such as cancers. Recent studies have demonstrated that the expression profiles of miRNAs are dysregulated in many diseases, including cancer, cardiovascular illnesses, infectious diseases, diabetes, neurodegenerative diseases, autism, autoimmune disorders, traumatic brain injury, and depression [2,3,4,5,6,7,8,9,10,11]. For these reasons, miRNAs have the potential to serve as useful biomarkers for early detection of cancer and monitoring patient treatment outcomes [12,13,14]. Therefore, rapid detection and accurate quantification of miRNA levels are of considerable clinical significance. Traditional approaches to miRNA detection include Northern blot, microarray, and quantitative reverse transcriptase (qRT)-PCR [15,16]. These methods are often time-consuming, laborious, and require skilled personnel and expensive equipment. Recently, there has been a growing interest in developing alternate nanotechnology-based methods for miRNA detection [17,18]. Biosensing strategies based on metal nanoparticles, such as gold and silver, have been widely studied due to their high sensitivity, low cost, and simple procedures [19,20]. Wang et al. developed a scheme to detect miRNA using the fluorescence quenching of gold nanoparticles [21]. Miao et al. proposed a colorimetric method for miRNA analysis based on hybridization chain reaction using silver nanoparticles [22]. However, these systems still require laboratory-based equipment (e.g., spectrophotometer) for detection and analysis, making them impractical for point-of-care. 

Smartphones have recently gained great interest as biomedical detection tools due to their widespread availability, simplicity of use, and increasing computational power. They provide a potent alternative to traditional laboratory-based analytical instrumentation, which is generally bulky and costly, and often requires a separate computer to function and skilled personnel to operate. Much of the data acquisition and processing operations can be performed and automated using a mobile application (app) accessed directly via a smartphone. To acquire data, the smartphone’s own internal sensor system can be leveraged; alternatively, the smartphone can be connected to external sensors [23]. The internal sensor commonly used for biosensing purposes is the smartphone’s built-in camera. The camera serves as a two-dimensional optical detector that can be utilized for microscopy imaging and for color-, fluorescence- and luminescence-based assays [24,25,26]. Smartphone-based colorimetric devices have been shown to operate using different sampling modalities and assay platforms such as solutions [27,28], test strips [29,30], and microfluidic chips [31,32]. These devices have been used to detect a wide variety of biomedical targets such as vitamin-D [33], cholesterol [34], cortisol [35], saliva alcohol [36], and proteins [37]. Despite the growing use of smartphones for biomedical detection, there are very few studies devoted to the detection of nucleic acid biotargets. Priye et al. reported a biochemical analysis platform on a drone that could perform polymerase chain reaction (PCR)-mediated DNA target detection using a smartphone [38]. Manusco et al. developed a smartphone accessory that could detect Kaposi Sarcoma associated with herpes virus nucleic acids using a colorimetric nanoparticle assay [39]. As smartphone-based detection platforms offer the potential for medical diagnostics at a global level due to their widespread accessibility, there is a need to further explore their role as practical biosensors for point-of-care and global health applications in today’s clinical landscape. 

In this work, we report a smartphone-based biosensing device for the detection and quantification of miRNA biomarkers. The device, called Krometriks, consists of a smartphone equipped with a 3D printed accessory and an integrated custom-built software. A silver nanoparticle (AgNP)-based assay, referred to as plasmonic coupling interference (PCI), developed by our group for multiplex nucleic acid biomarker detection using surface-enhanced Raman scattering (SERS) was adapted for colorimetric sensing [40,41]. When metallic nanoparticles aggregate, due to an effect known as plasmonic coupling, their absorbance and scattering pattern changes, leading to a change in color and absorption spectra [42]. This optical behavior exhibited by nanoparticles has been utilized in the colorimetric detection of metal ions and biomolecules [43,44]. In our assay, the extent of nanoparticle aggregation in solution is determined by the number of miRNA targets present in the sample. miRNA quantification is achieved by measuring the color change of the solution. We demonstrate the proof of concept of our device with synthetic miRNA 21 (miR-21) as the target molecule. miR-21 is selected in this study as it is an important biomarker that has been shown to be dysregulated in different types of cancer (e.g., breast, colon, lung) and various non-cancer diseases, such as cardiovascular, infectious, and neurological diseases [3,4,5,45,46,47]. We demonstrate that Krometriks can measure nanomolar concentrations of the miRNA target requiring very small amounts (100 µL) of a sample. The detection accuracy of Krometriks is shown to be comparable to that of a laboratory-based benchtop spectrophotometer. Krometriks is portable, simple to use, and can perform automated analysis. The system has the potential to be a useful colorimetric tool for clinically diagnostics for point-of-care and global health applications in low-resource settings.

## 2. Methods

### 2.1. Device Design

**Figure 1:**
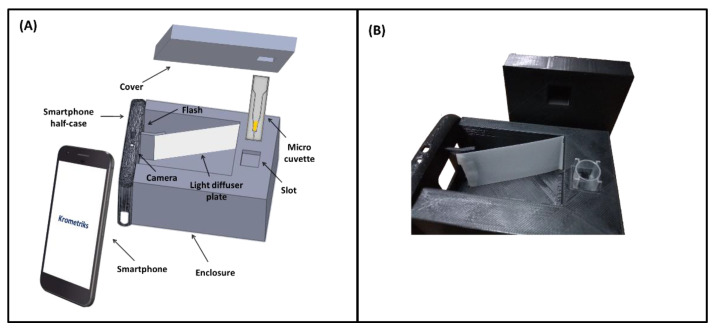
(**A**) 3-D schematic diagram of the Krometriks system and its components. The smartphone’s camera and flashlight act as the sensing detector and light source, respectively. (**B**) Picture of the 3-D printed Krometriks accessory.

The Krometriks device consists of a smartphone equipped with a 3D printed accessory and a custom mobile app. Although the smartphone used for this study is a Galaxy S6 (Samsung, Suwon-si, South Korea), the detection modality can be applied to other smartphone platforms. The phone’s flashlight provides the illumination, and the built-in camera serves as the optical sensing unit. The smartphone accessory was designed using a 3D modeling software (SolidWorks, Dassault Systèmes) and fabricated using 3D printing. The accessory is comprised of the top half of the smartphone case joined to a specifically designed enclosure (90 mm × 69 mm × 59 mm) with its cover. The half-case holds the smartphone while the enclosure has a slot to hold a micro-cuvette (BrandTech Scientific, Inc., Essex, CT, USA). The slot is kept at a specific distance (75 mm) from the camera lens. To obtain uniform illumination of samples with a diffuse beam of the phone’s flashlight, the enclosure also contains a two-part opaque-translucent light diffuser plate. The shorter opaque part is a 3D-printed polymer plastic, and the longer translucent part is a polyethylene terephthalate (PET) diffuser film (Inventables, Chicago, IL, USA). The inside rear wall of the enclosure is affixed with white paper that serves as the background screen. The enclosure is tightly covered to prevent external light from interfering with measurements. Figure 1A depicts a schematic diagram of the Krometriks system. Before each measurement, the micro-cuvette containing the sample is placed in the slot, and the enclosure is covered. Figure 1B shows a picture of the Krometriks enclosure (with the micro-cuvette placed in its slot) and the cover. A custom smartphone app is programmed to acquire and analyze the image data. 

### 2.2. Smartphone Software

To manage the process flow of the Krometriks device, a custom software application (app) was developed in our laboratory using an integrated development environment (Android Studio, Google) for the Android OS. The app is capable of image acquisition, data preprocessing, data treatment, result display, and data storage in an automated fashion. After the sample is placed in the enclosure slot and covered, the app is accessed for measurements. The app offers options to create a new calibration curve or to use a previously established curve in order to perform sample analysis. 

#### 2.2.1. Calibration 

**Figure 2:**
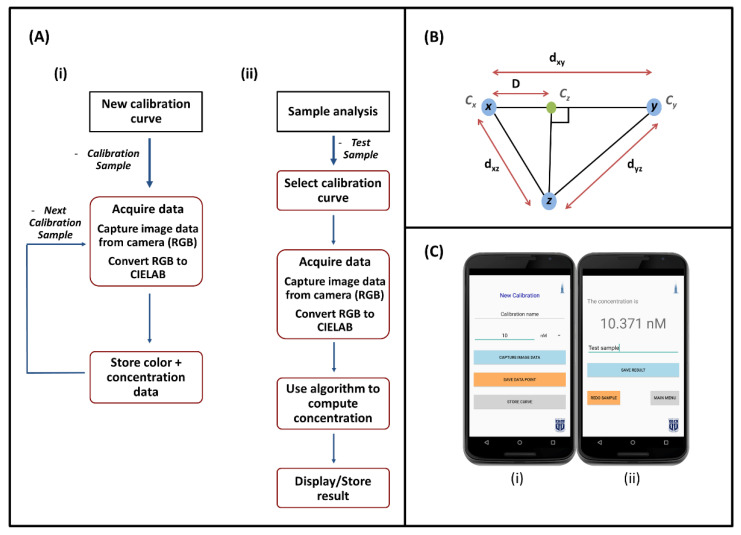
(**A**) Schematic diagram of the process flow for (**i**) creating a new calibration curve, and (**ii**) analyzing a test sample using the Krometriks app. (**B**) 2-D representation of the geometric relationship between *x*, *y*, and *z*, where *z* is the test sample point, *x* is the calibration points closest to *z*, and *y* is the calibration point on either side of *x* in a sorted list of calibration points that are closest to *z*. The concentration *C_z_* is linearly interpolated on the line between *C_x_* and *C_y_*. (**C**) Screenshots from the Krometriks app showing the page: (**i**) for adding a new data point while creating a new calibration curve, (**ii**) displaying the result-estimated concentration value of test sample.

A set of reference samples with known concentrations of the target analyte is used to establish a calibration curve before performing actual analyses. To create a new calibration curve, the corresponding option is selected on the app. The calibration name is entered, followed by data acquisition of the first calibration sample. The sample concentration value is entered, and the image data is subsequently captured. A small red rectangle (20 × 40 pixels) on the camera preview indicates the region of interest, which is set to the center of the sample. The pixels from the region of interest are only considered while computing the color of the sample. A reference region (35 × 35 pixels), indicated by a blue square over the white background screen on the camera preview, is also considered in the calculations to account for variations between measurements. The app captures color data from multiple images of the sample. The pixel data acquired by the smartphone is in the RGB color space, and each pixel consists of three channels (red, green, blue). The pixels are in a 24-bit color system, each channel having 8 bits and an intensity value between 0–255 (2^8^ = 256). Following the capture phase, the pixel values in the region of interest are averaged over the multiple images to get a single color RGB value for the sample. This color value is then converted to the CIELAB color space. The data acquisition process is performed for each calibration sample. Finally, the calibration data is sorted according to the concentration values and stored in the phone’s internal storage. The entire process is schematically depicted in Figure 2A(i). 

#### 2.2.2. Color Space Conversion

The CIELAB color space has three channels represented by L*, a*, and b*. L* represents the lightness, with values ranging from 0 (denoting black) to 100 (denoting white). The a* channel represents the magenta-green spectrum position with negative values indicating green and positive values indicating magenta. The b* channel represents the position on the blue-yellow spectrum, positive values denoting yellow and negative values denoting blue. Conversion from RGB to CIELAB space involves the following four steps [48,49]:

1. The RGB value (*R*, *G*, *B*) is normalized (*R_n_*, *G_n_*, *B_n_*) to a value in the range [0, 1]. 

2. The normalized non-linear RGB values are linearized (*R_l_*, *G_l_*_,_ *B_l_*) with the following equation:

$$C_{l}=\left\{\begin{array}{cc} \;{\left(\frac{C_{n}+0.055}{1.055}\right)}^{2.4} & :\;\;C_{n}>0.04045\\ \frac{C_{n}}{12.92} & :\;C_{n}\leq 0.04045 \end{array}\right\}$$

where C∈{R, G, B}

3. The linearized. RGB values are converted to tristimulus values (*X*, *Y*, *Z*) under standard illuminant D65 through these three associations:

$$\begin{array}{l} X=0.4124\;R_{l}+0.3576\;G_{l}+0.1805\;B_{l}\\ Y=0.2126\;R_{l}+0.7152\;G_{l}+0.0722\;B_{l}\\ Z=0.0193\;R_{l}+0.1192\;G_{l}+0.9505\;B_{l} \end{array}$$

4. The tristimulus values (*X*, *Y*, *Z*) are multiplied by a factor of 100 and then used to compute the CIELAB values (L*, a*, b*) using the following equations:

$$\begin{array}{c} L^{*}=116\;f\left(\frac{X}{X_{n}}\right)-16\\ a^{*}=500\left(f\left(\frac{X}{X_{n}}\right)-f\left(\frac{Y}{Y_{n}}\right)\right)\\ b^{*}=200\;\left(f\left(\frac{Y}{Y_{n}}\right)-f\left(\frac{Z}{Z_{n}}\right)\right) \end{array}$$

where

f(x)={x3 : x>(629)3(841108)x+429 : x≤(629)3}

and *X_n_* = 95.047, *Y_n_* = 100 and *Z_n_* = 108.883 are the tristimulus values of the white point of standard illuminant D65 using the 2° standard observer and normalized for relative luminance.

#### 2.2.3. Sample Analysis

Prior to choosing the option for sample analysis, the corresponding calibration curve is selected from the list of stored calibration curves on the app. Image acquisition and color determination for the test sample follow the process described previously for calibration samples. Figure 2A(ii) depicts the schematic diagram of the process. The following algorithm is then used to quantify the concentration of the test sample:

1. The test point is compared to each data point in the stored calibration curve. Comparison takes place by calculating the color difference between the two points. The color difference is calculated by taking the Euclidean distance between the color points as per the following equation:

$$d_{12}=\sqrt{{\left(L_{2}^{*}-L_{1}^{*}\right)}^{2}+{\left(a_{2}^{*}-a_{1}^{*}\right)}^{2}+{\left(b_{2}^{*}-b_{1}^{*}\right)}^{2}}$$

where L_1_*, a_1_*, b_1_* and L_2_*, a_2_*, b_2_* are the CIELAB components of the color points, respectively. The distance between each calibration point and the test point is calculated and the calibration point corresponding to the smallest distance is noted. Let this point be *p_n_*, where *n* is the index of the point in the list of sorted calibration points.

2. The distance between the test point and calibration points *p_n−1_* and *p_n+1_* is calculated and the smaller of the two is noted (*p_n−1_* and *p_n+1_* are the two adjacent points to *p_n_* on the sorted calibration. If p_n_ is the first or the last point on the calibration, then the sole adjacent point is noted). Let this point be represented by *y*, *p_n_* by *x* and the test point by *z* henceforth.

3. Let the concentrations corresponding to *x*, *y*, and *z* be denoted by *C_x_*, *C_y_*_,_ and *C_z_*_,_ respectively. A linear gradient is assumed between the successive calibration points. Figure 2B shows a schematic visual representation of the process. Interpolating between x and y, the point on the line joining *x* and *y* which is closest to point *z* is located and its distance to point *x* (denoted by *D*) is calculated using the equation:

$$D=\frac{d_{xy}^{2}+d_{xz}^{2}-d_{yz}^{2}}{2d_{xy}}$$

4. The test sample concentration *C_z_* is calculated using the equation:

$$C_{z}=C_{x}+\frac{\left(C_{y}-C_{x}\right)D}{d_{xy}}\;$$

### 2.3. Nanoparticle Assay Based on Plasmonic Coupling Interference

**Figure 3:**
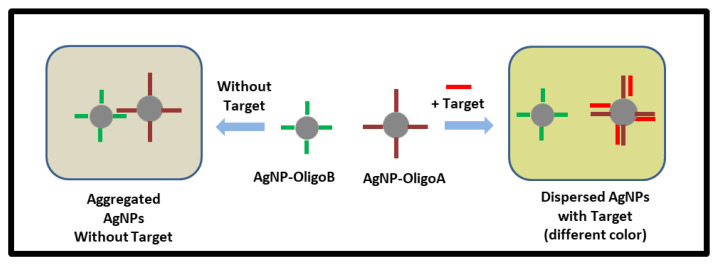
Schematic diagram showing the operating principle of the colorimetric sensing method using nanoparticle-base assay. Left: Aggregated AgNPs without target. Right: Disperesed AgNPs with target.

Our laboratory has been developing various nanosystems using surface-enhanced Raman scattering (SERS) for a wide variety of applications spanning chemical sensing, biomedical diagnostics, and artwork identification [50,51,52,53]. We have previously developed the PCI method as a silver nanoparticle-based assay using SERS detection [40,41]. Although the SERS detection method provides narrow-band Raman spectra most appropriate for multiplex nucleic acid biomarker monitoring, it requires the use of excitation lasers, high-resolution spectrometers, and sensitive detectors. Here, the PCI method is adapted to take advantage of the color change involved in the assay in order to use simple smartphone-based platforms for detection. The smartphone-based detection system is most suitable for point-of-care applications in low-resource settings. The operating principle of the plasmonic nanoparticle-based detection method described in previous works is schematically shown in Figure 3. This approach involves two nanoprobe constructs, probe-A and probe-B, prepared for one specific miRNA sequence. Probe-A is designed to be complementary to a specific miRNA sequence as a capture probe. To induce aggregation, probe-B is designed to have sequences complementary to probe-A, leading to nanoparticle aggregation (Figure 3, left). When metallic nanoparticles aggregate, due to the effect known as plasmonic coupling, their absorbance and scattering pattern change and exhibit a specific color and absorption spectra. Our approach then utilizes the target sequences as competitors of the probe-B in a competitive binding process; this process interferes with the plasmonic coupling effect. As a result, the aggregate formation of nanoparticles is disrupted by the target molecules (Figure 3, right), resulting in a shift of the plasmon resonance band, leading to sample color changes depending on the aggregate size.

### 2.4. Sample Preparation 

The sample preparation process was adapted from the methods described in previous works [40,41]. Briefly, silver nanoparticles were prepared and conjugated to two oligonucleotide sequences (oligoA and oligoB) specific to miR-21 (see Supplementary Information for more details). Final samples were prepared by mixing AgNP-oligoA and AgNP-oligoB nanoprobes in a 1:1 volume ratio with the synthetic miR-21 target (5′-TAGCTTATCAGACTGATGTTGA-3′). The mixture was incubated in a 10 mM Tris-HCl buffer (pH 8.0) solution containing 0.15 M sodium chloride, 2.5 mM magnesium chloride, and 0.01% Tween 20 (total volume: 100 µL) for 60 min. Following incubation, 10 µM of a custom stopper sequence (5′-TAGCTTATCAGAC-3′) was added to stop the reaction between oligoA, oligoB and the target for a few hours and the samples were measured.

### 2.5. Statistical Analysis

Comparison of the estimation accuracy of Krometriks and the spectrophotometer was performed by calculating and comparing the estimation error (i.e., a large error corresponds to a low accuracy) using the following equations:

$$Absolute\;Percentage\;Error\left(APE\right)=\left|\frac{Actual-Estimated}{Actual}\right|\;\times \;100\;Mean\;Absolute\;Percentage\;Error\left(MAPE\right)=\frac{1}{n}\overset{n}{\underset{i=1}{\sum }}APE_{i}\;$$

where *Actual* is the actual value of the sample, *Estimated* is the estimated value of the sample, and *n* is the total number of samples 

To determine the difference in estimation accuracy between two methods, a statistic test based on the two proportion *Z*-test [54] was performed using the following equation:

$$Z=\frac{MAPE_{2}-MAPE_{1}}{\sqrt{2*MAPE_{avg}\left(100-MAPE_{avg}\right)/n}}$$

where

$$MAPE_{avg}=\frac{\;MAPE_{1}+MAPE_{2}}{2}$$

and *MAPE*_1_ and *MAPE*_2_ are the mean absolute percentage error of method 1 and method 2, respectively, and *n* is the number of samples.

Based on a standard normal distribution with a 5% level of significance, if *Z* < −1.645, then we can say with 95% confidence that method 2’s estimation accuracy is more than that of method 1. If *Z* > 1.625, then we can say with 95% confidence that method 1′s estimation accuracy is more than that of method 2. Otherwise, if −1.625 < *Z* < 1.625, then there is no significant difference (for a 5% level of significance) in the estimation accuracy between method 1 and method 2. 

## 3. Results and Discussion

As the diagnostic systems are often designed to be operated in the field under ambient light conditions, an important consideration for smartphone-based optical detection systems involves the proper handling of the effect of surrounding light. Devices using ambient light for image data acquisition [55] are subject to the inherent variability in the surrounding conditions between and during measurements. This issue has been addressed by using signal normalization with reference color areas [56] and by enclosing the sample in an enclosure to prevent outside light from entering the sample compartment [57]. In addition to performing normalization using a reference region, Krometriks also uses a custom-built 3D-printed enclosure to hold samples. This accessory holds samples in a fixed position with respect to the camera lens and inhibits relative movements between the smartphone and samples between measurements. The Krometriks’ accessory is intentionally designed to be simple and easy to use. Some smartphone-based systems contain various optical and electrical parts as components of the accessory [58,59]. These complex components are challenging to maintain and repair, making them impractical candidates for point-of-care and field applications. The Krometriks accessory has a simple design and consists of only 3D-printed parts, a diffuser paper, and a white paper. It is low-cost and easy to maintain and repair, as the diffuser paper and white paper can be quickly replaced, if needed. Inexpensive disposable micro-cuvettes are used for sample measurements. All these design considerations have been made such that Krometriks can be used for field applications and in areas with limited resources. With slight changes to the accessory design, Krometriks can be made compatible with different types of smartphone models and specifications. 

The Krometriks’s operation is managed using a custom-designed mobile app created for the Android platform. The app has an easy-to-use interface that can be conveniently maneuvered using basic smartphone literacy. Before each data capture session, the image is autofocused by the app to get the best possible image resolution. Sample analysis (data acquisition to result display) only takes a few clicks and a few minutes of the user’s time, providing a rapid output with minimal user input. The data processing algorithm requires the color of the sample to be converted from RGB to CIELAB space. Since changes in the RGB space are non-linear in nature, it is not the best suited for measuring color differences. The CIELAB color space is chosen as it is device-independent and perceptually uniform. Each color can be represented by a point in three-dimensional Euclidean space with L*, a*, and b* as the three coordinates. Changes in these coordinates are uniform with changes in the perceived color, and this representation defines color difference as the Euclidean distance between the points. Our data processing algorithm using the CIELAB color scheme is independent of any correlations with the miRNA target being measured, and in the future, this algorithm has the potential to be used with other types of assays. 

In this work, Krometriks is used for the colorimetric miRNA analysis of a nanoparticle-based assay previously developed by our group [40,41]. As discussed previously, the operating principle of the bioassay is based on the hybridization process of nucleic acids, leading to nanoparticle aggregation: when mixed, AgNP-oligoA and AgNP-oligoB nanoprobes hybridize with each other (oligoA and oligoB are complementary sequences), leading to nanoprobe aggregation. To detect a target of interest, the AgNP-oligoA is designed to be fully complementary to the target sequence. Thus, the presence of the target strand acting as a competitor to the AgNP-oligoB nanoprobes in a competitive binding process inhibits nanoprobe aggregation. The extent of the hybridization-mediated aggregation determines the color of the solution and the profile of the absorption spectrum. The target concentration can be estimated by measuring the absorbance (spectrophotometer) or by quantifying the color of the sample solution (Krometriks) and fitting the data to a calibration curve. 

**Figure 4:**
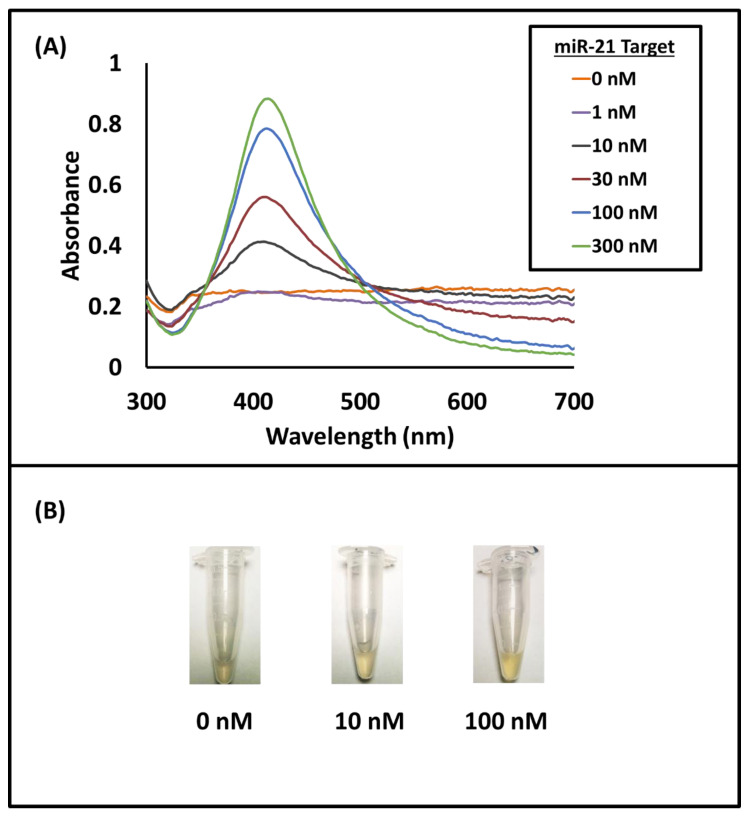
(**A**) Absorption spectra of samples with different miR-21 target concentrations (0–300 nM). An increase in target concentration results in an increase in peak intensity around 412 nm and a decrease around 650 nm. (**B**) Pictures of sample solutions with 0 nM, 10 nM, and 100 nM of miR-21 target. A larger amount of miR-21 target results in a stronger yellow color of the sample solution.

To investigate the effect of miR-21 target addition on nanoprobe aggregation, samples mixed with different amounts of the miR-21 target were measured with a spectrophotometer (FLUOstar Omega, BMG LABTECH) in the UV-Vis region. The results were compared with data obtained with the much smaller Krometriks system. In the presence of increasing miR-21 target concentrations, an increase in the absorbance of the plasmon resonance peak of isolated AgNPs at around 412 nm was observed. In addition, a decrease in the absorbance around 650 nm for aggregated AgNPs was observed (Figure 4A for absorbance spectra). The sample solution turned more yellow from greyish yellow with increasing target concentration (Figure 4B), indicating that more AgNP-nanoprobe aggregation occurred in the absence of the target miRNAs. The nanoprobes were well-dispersed or had properly aggregated (depending on the target amount) as the yellow color of the solution did not change over time. To quantify the colorimetric assay, the total absorbance (blank subtracted) difference was calculated by subtracting the absorbance peak intensity at 650 nm from that at 412 nm. The blank sample in this case was the solution containing only the nanoprobes AgNP-oligoA and AgNP-oligoB and no target molecules. 

**Figure 5:**
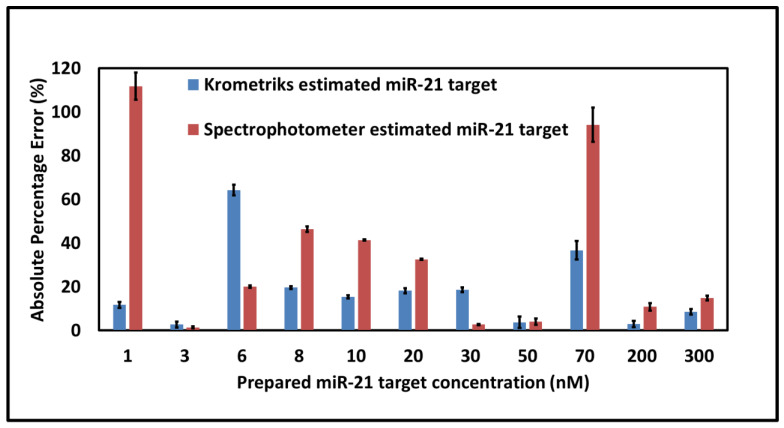
Bar plot showing the absolute percentage errors for the estimated concentration values obtained by the Krometriks app (blue) and the spectrophotometer (red) for different miR-21 target concentrations. The error bars indicate one standard deviation of variation in the measurement values.

The utility of Krometriks was demonstrated by comparing its colorimetric detection accuracy to that obtained with a laboratory-based benchtop spectrophotometer. First, calibration curves were generated for both modes of measurement (Krometriks and spectrometer). The same set of calibration samples containing different miR-21 target amounts was used. For the Krometriks device, the calibration curve was established using the process described in the Section 2. The detailed data of the calibration curve were stored internally in the app. For the spectrophotometer, the calculated absorbance difference (between the peak intensity at 412 nm and 650 nm) exhibited a linear relationship with the target concentration (on the log scale). A linear fit to the calibration data with a R^2^ = 0.962 was used as the spectrophotometer-generated calibration curve (Figure S1 in the Supplementary Information). A set of test samples was then analyzed using the Krometriks’ algorithm (described in the Section 2) as well as by fitting the test data to the spectrophotometer-generated calibration curve. Figure 5 shows a bar plot of the absolute percentage error (APE) for the estimated concentrations of different miR-21 targets obtained using the Krometriks device (blue) and the spectrophotometer (red). Each APE value (calculated and discussed in the Section 2) for the Krometriks device and the spectrophotometer was an average of three measurements, respectively. The error bars indicated one standard deviation from the mean. As depicted in the plot, Krometriks provided equally good or better estimations than the spectrophotometer. To evaluate and compare the overall accuracy of the two methods, the mean absolute percentage error (MAPE) for both methods was calculated (discussed in the Section 2). The MAPE was 18.411% for Krometriks and 34.57% for the spectrophotometer. The lesser MAPE value for Krometriks concurs with the inference from the bar plot that the accuracy of Krometriks is as good or better than that of the spectrophotometer. A test of statistical significance was performed on these MAPE values based on a modified two-proportion *Z*-test (described in the Section 2). This gave a *Z* value of −0.859, indicating that the difference in the accuracy of Krometriks and the spectrophotometer was not significant (for a 5% level of significance). Therefore, the Krometriks device provided accurate quantitative estimations, demonstrating a performance comparable to the spectrophotometer. All the analyses were automated on the Krometriks app, and no additional intervention of the user was required after sample measurement to get the results. It is noteworthy that the spectrophotometer required the user’s additional efforts to calculate the estimated concentrations requiring more effort and time in comparison to Krometriks. Each sample from measurement to result display, including data analysis, took less than 1 min to accomplish with Krometriks and 30 min to accomplish with the spectrophotometer. Our results showed that Krometriks could detect miR-21 targets with concentrations ranging from 1 nM to 300 nM requiring only 100 µL of solution volume. On the other hand, RT-qPCR typically requires around two to three hours for a single run [60,61], laboratory-based equipment, and skilled personnel to operate sample amplification; thus, it is not suitable for use at the point-of-care setting with limited resources. The advent of more sensitive nanoparticle-based assays for miRNA detection and its integration with platforms like Krometriks holds promise for more precise and accessible diagnostics in the future. 

## 4. Conclusions

There is a strong need to develop simple and practical biosensing devices for use in point-of-care settings and underserved areas with limited resources. MicroRNA have been shown to serve as important biomarkers of a wide variety of illnesses, ranging from cancers and cardiovascular illnesses to infectious diseases [2,3,4,5,6,7,8,9,10,11]. Furthermore, miRNAs are also useful biotargets in non-medical application areas such as plant biology and renewable biofuel research [62,63]. Advances in nanotechnology-based miRNA detection schemes have made the process simpler and more inexpensive, but laboratory equipment is still required for measurements and analysis. The strong computing power and ease of usability of smartphones can be harnessed to create simple and portable diagnostic platforms that can offer suitable alternatives to traditional laboratory equipment. In this work, we present a smartphone-based device, Krometriks, that performs rapid automated colorimetric miRNA biomarker detection. We show that Krometriks could detect miR-21 target down to nanomolar concentrations, required only 100 µL of sample volume, and provided a result in less than a minute. We also show that Krometriks performs comparably to a benchtop spectrophotometer in detection accuracy, with the added advantages of simplicity, portability, result automation, and cost-effectiveness. Systems like Krometriks offer a useful colorimetric tool for accessible and affordable miRNA diagnostics, especially for point-of-care and global health applications. 

## Supplementary Materials

The following are available online at https://www.mdpi.com/article/10.3390/s21238044/s1, Figure S1: Plot showing the absorbance difference (A_412_–A_650_) of the calibration samples with the log_10_ of their miR-21 target concentration on the x axis. Each measurement was performed thrice. The error bars show one standard deviation of variation in the measurements. The linear fit of the data (R^2^ = 0.962) was used as the calibration curve for calculating the concentration of the test samples.
