# Supplementary material for: Smartphone-Based Device for Colorimetric Detection of MicroRNA Biomarkers Using Nanoparticle-Based Assay
Source: Sensors (Basel). 2021 Dec 1;21(23):8044. doi: 10.3390/s21238044 (PMC8659705; doi:10.3390/s21238044)
Supplement: Supplementary file 1 [file sensors-21-08044-s001.zip › sensors-1462930-supplementary.pdf]

Supplementary Information

Smartphone-Based Device for Colorimetric Detection of microRNA Biomarkers Using Nanoparticle-Based Assay

Tushar Krishnan [1,2], Hsin-Neng Wang [1,2] and Tuan Vo-Dinh [1,2,3,*]

1 Fitzpatrick Institute for Photonics, Duke University, Durham, NC 27708, USA; tusharkrishnan@gmail.com (T.K.); hsinneng.wang@duke.edu (H.-N.W.)
2 Department of Biomedical Engineering, Duke University, Durham, NC 27708, USA
3 Department of Chemistry, Duke University, Durham, NC 27708, USA
* Correspondence: tuan.vodinh@duke.edu

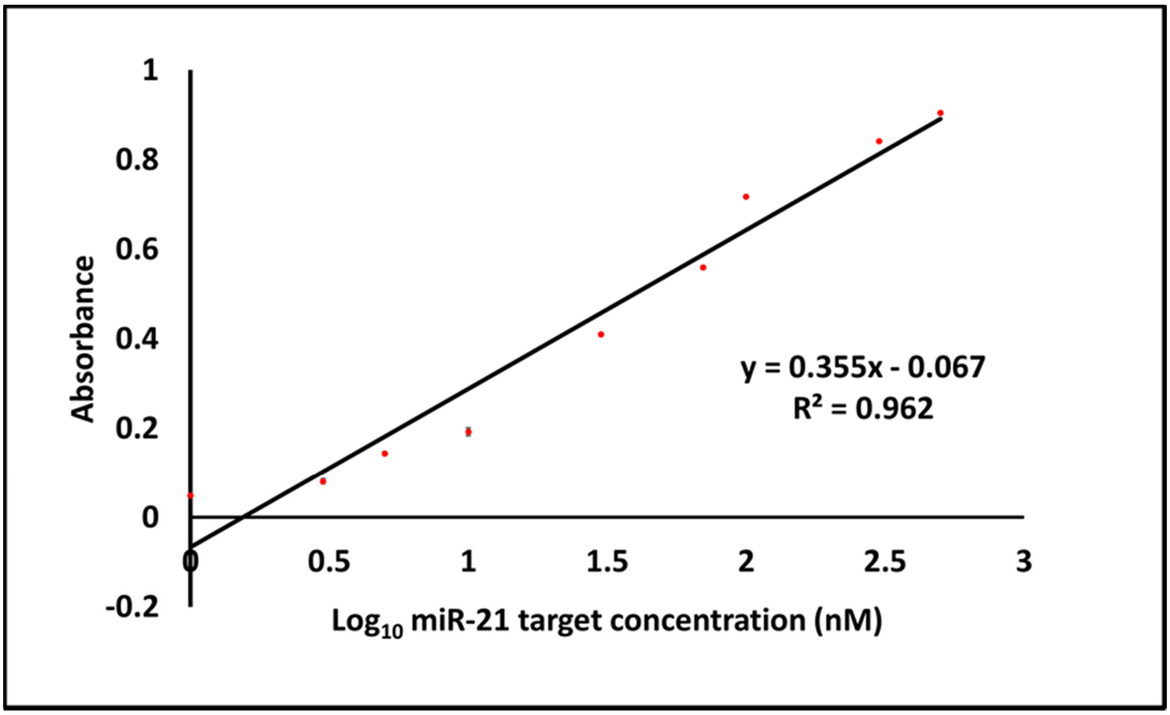

Figure S1. Plot showing the absorbance difference ($A_{412} – A_{650}$) of the calibration samples with the log_{10} of their miR-21 target concentration on the x axis. Each measurement was performed thrice. The error bars show one standard deviation of variation in the measurements. The linear fit of the data ($R^2 = 0.962$) was used as the calibration curve for calculating the concentration of the test samples.

Sample Preparation

Sample preparation was adapted from the methods described in previous works [40, 41].

Preparation of silver nanoparticles

Silver nanoparticles were prepared by swiftly adding 1 mL of a solution containing 15 mM hydroxylamine hydrochloride and 30 mM sodium hydroxide to 9 mL of 1.1 mM silver nitrate solution under vigorous stirring conditions for 1 hr. The colloidal solution was kept at 4°C and used within a few weeks.

Conjugation of oligonucleotide to silver nanoparticles

Silver nanoparticles were incubated with 0.4 μM thiolated DNA oligonucloetides (oligoA having the sequence 5′-SH- TCAACATCAGTCTGATAAGCTA-3′ and oligoB having the sequence 5′-SH-TAGCTTATCAGAC-Cy3-3′) in 0.25 mM magnesium chloride solution (total volume: 1mL) overnight at room temperature. To stabilize the solution, a mixture of 100μM mPEG-SH and 0.5μM mPEG-SH (MW 5000) was added for 10 min followed by the addition of 0.01% Tween 20. The functionalized nanoparticles were washed thrice with 10 mM Tris-HCl buffer (pH 8.0) containing 0.01% Tween 20 using repeated centrifugation at 12,000 rpm for 10 min. The purified nanoprobes were suspended in 1 mL of 10 mM Tris-HCl buffer (pH 8.0) containing 0.01% Tween 20 and kept at 4°C.

Materials

Silver nitrate (99.995%) was procured from Alfa Aesar (Ward Hill, MA). Hydroxylamine hydrochloride and sodium hydroxide (pellet) were purchased from Avantor Performace Materials (Phillipsburg, NJ). SH-PEG-COOH (O-(2-Carboxyethyl)-O′-(2 mercaptoethyl)-heptaethylene glycol), Tris-HCl buffer (1 M, pH 8.0), magnesium chloride (1M) and sodium chloride (5M) were purchased from Sigma-Aldrich. mPEG-SH (MW 5000) was purchased from Nanocs, Inc. (Boston, MA) and Tween 20 (10%) was purchased from G-Biosciences (St. Louis, MO). All DNA oligonucleotides were purchased from Integrated DNA Technologies, Inc. (Coralville, IA).
